# Protease-activated receptor 1 in diabetes and its chronic complications: signaling mechanisms and emerging therapeutic advances

**DOI:** 10.3389/fphar.2026.1891733

**Published:** 2026-08-25

**Authors:** Yanrui Wei, Bing Wang, Qiuyue Wang

**Affiliations:** Department of Endocrinology and Metabolism, The First Affiliated Hospital of China Medical University, Shenyang, Liaoning, China

**Keywords:** biased signaling, diabetes mellitus, diabetic chronic complications, protease-activated receptor 1, targeted therapy

## Abstract

Diabetes mellitus (DM) is a systemic metabolic disorder characterized by chronic hyperglycemia, which can affect the kidneys, retina, peripheral nerves, and cardiovascular system, ultimately leading to multiple chronic complications. Protease-activated receptor 1 (PAR1) is a G protein-coupled receptor activated through canonical cleavage, such as thrombin-mediated cleavage, and non-canonical cleavage, such as cleavage by activated protein C or matrix metalloproteinase-1. These activation modes initiate diverse downstream signaling pathways, including Gαq/PLC-β/PKC, Gα12/13/Rho, and β-arrestin-dependent signaling, and contribute to inflammation, fibrosis, angiogenesis, and apoptosis. This review summarizes the role of PAR1 in the pathogenesis of type 2 diabetes mellitus (T2DM), focusing on its effects on endothelial insulin resistance, adipose tissue inflammation, and brown adipose tissue dysfunction. We further discuss the pathogenic mechanisms of PAR1 in diabetic kidney disease, diabetic retinopathy, diabetic peripheral neuropathy, and diabetic cardiovascular disease. This review also outlines recent advances in PAR1-targeted therapies, including orthosteric antagonists such as vorapaxar, biased signaling modulators such as 3K3A-APC and parmodulins, and intracellular inhibitors such as PZ-128. Their therapeutic potential and current challenges in antithrombotic therapy and target-organ protection are also discussed, with the aim of informing more precise strategies for preventing and treating DM and its chronic complications.

## 1 Introduction

Diabetes mellitus (DM) is a multifactorial metabolic disorder characterized by chronic hyperglycemia. Its pathogenesis mainly involves reduced insulin sensitivity in peripheral tissues and/or impaired insulin secretion due to pancreatic β-cell dysfunction. Persistent hyperglycemia can cause multi-organ damage and dysfunction, particularly in the eyes, peripheral nerves, vasculature, kidneys, and heart. Protease-activated receptor 1 (PAR1) was the first identified member of the protease-activated receptor family, a subfamily of G-protein-coupled receptors (GPCRs). It was originally identified as the thrombin receptor on platelets and, upon activation, drives platelet aggregation and hemostasis. Under physiological conditions, PAR1 helps maintain vascular endothelial barrier integrity, regulate basal hemodynamics, and support normal hemostasis. Under pathological conditions, such as vascular injury, severe infection, or metabolic dysregulation, PAR1 can become excessively or aberrantly activated. This activation contributes to apoptosis, pro-inflammatory cytokine release, vascular barrier disruption, pathological platelet activation, and tissue fibrosis, thereby contributing to cardiovascular, inflammatory, and metabolic diseases (Willis Fox and Preston, 2020; Ossovskaya and Bunnett, 2004). Increasing evidence links PAR1 to the development of DM and its chronic complications, suggesting that PAR1 may serve as a therapeutic target. Compared with other established pathways involved in diabetic complications, PAR1 has several distinctive features as a therapeutic target. PAR1 can be activated by multiple pathological stimuli and may link hyperglycemic stress, hypercoagulability, and chronic inflammation in DM. Taken together, PAR1 blockade may interrupt a common downstream hub through which upstream injury signals promote target-organ fibrosis. In addition, the biased signaling properties of PAR1 allow either full inhibition, as with vorapaxar, or selective activation of protective pathways, as with APC-mimetic biased agonists. This signaling tunability makes PAR1 an attractive therapeutic target. This review summarizes the relationship between PAR1 and DM and its chronic complications, as well as recent progress in the development of PAR1-targeted therapies.

## 2 Methods

### 2.1 Search strategy and selection criteria

This narrative review was conducted in accordance with the methodological guidance for narrative syntheses to ensure transparency and reproducibility. A systematic literature search was performed in three major electronic databases: PubMed (including MEDLINE), Web of Science, and Scopus, covering the publication period from January 2000 to June 2026. The search strategy was developed by combining Medical Subject Headings (MeSH) and relevant free-text terms using Boolean operators (AND/OR). The core search strings were constructed as follows:

For PAR1: (“Protease-Activated Receptor 1”[MeSH] OR “PAR1” OR “F2R” OR “thrombin receptor” OR “coagulation factor II receptor”)
For Diabetes: (“Diabetes Mellitus” [MeSH] OR “diabetes” OR “T2DM” OR “hyperglycemia” OR “insulin resistance”)
For Complications: (“Diabetic Nephropathies” [MeSH] OR “Diabetic Retinopathy” [MeSH] OR “Diabetic Neuropathies” [MeSH] OR “Diabetic Cardiomyopathies” [MeSH] OR “Atherosclerosis” [MeSH] OR “chronic diabetic complications” OR “microvascular” OR “macrovascular”)

The final search syntax combined these three components as: (“Protease-Activated Receptor 1” OR “PAR-1” OR “PAR1”) AND (“Diabetes Complications” OR “Diabetic Nephropathy” OR “Diabetic Retinopathy” OR “Diabetic Neuropathies” OR “Cardiovascular Diseases” OR “Diabetic Cardiomyopathies” OR “Atherosclerosis” OR “Diabetic Angiopathies”). The search was limited to articles published in English to accommodate the linguistic expertise of the authors and the regional literature.

### 2.2 Inclusion and exclusion criteria

Studies were considered eligible for inclusion if they met the following criteria: (1) peer-reviewed original research (including *in vitro* mechanistic studies, animal models, and human clinical trials), high-quality systematic reviews, and meta-analyses; (2) investigations specifically addressing the role of PAR1 in the pathogenesis, progression, or treatment of diabetes mellitus or its associated complications (including kidney, retinal, neurological, and cardiovascular complications); and (3) studies with available full texts addressing PAR1-related signaling pathways (e.g., G-protein coupling, β-arrestin biased signaling, or receptor transactivation).

Articles were excluded based on the following criteria: (1) conference abstracts, editorials, letters, case reports (with fewer than 5 cases), or expert opinions lacking original data; (2) studies focusing solely on other PAR subtypes (PAR2, PAR3, PAR4) without mentioning PAR1; (3) studies on PAR1 in non-diabetic diseases (e.g., cancer metastasis or stroke unrelated to diabetes); without translational relevance to glucose metabolism or diabetic end-organ damage; and (4) duplicate publications or studies with irretrievable full texts.

### 2.3 Screening process and data extraction

**FIGURE 1:**
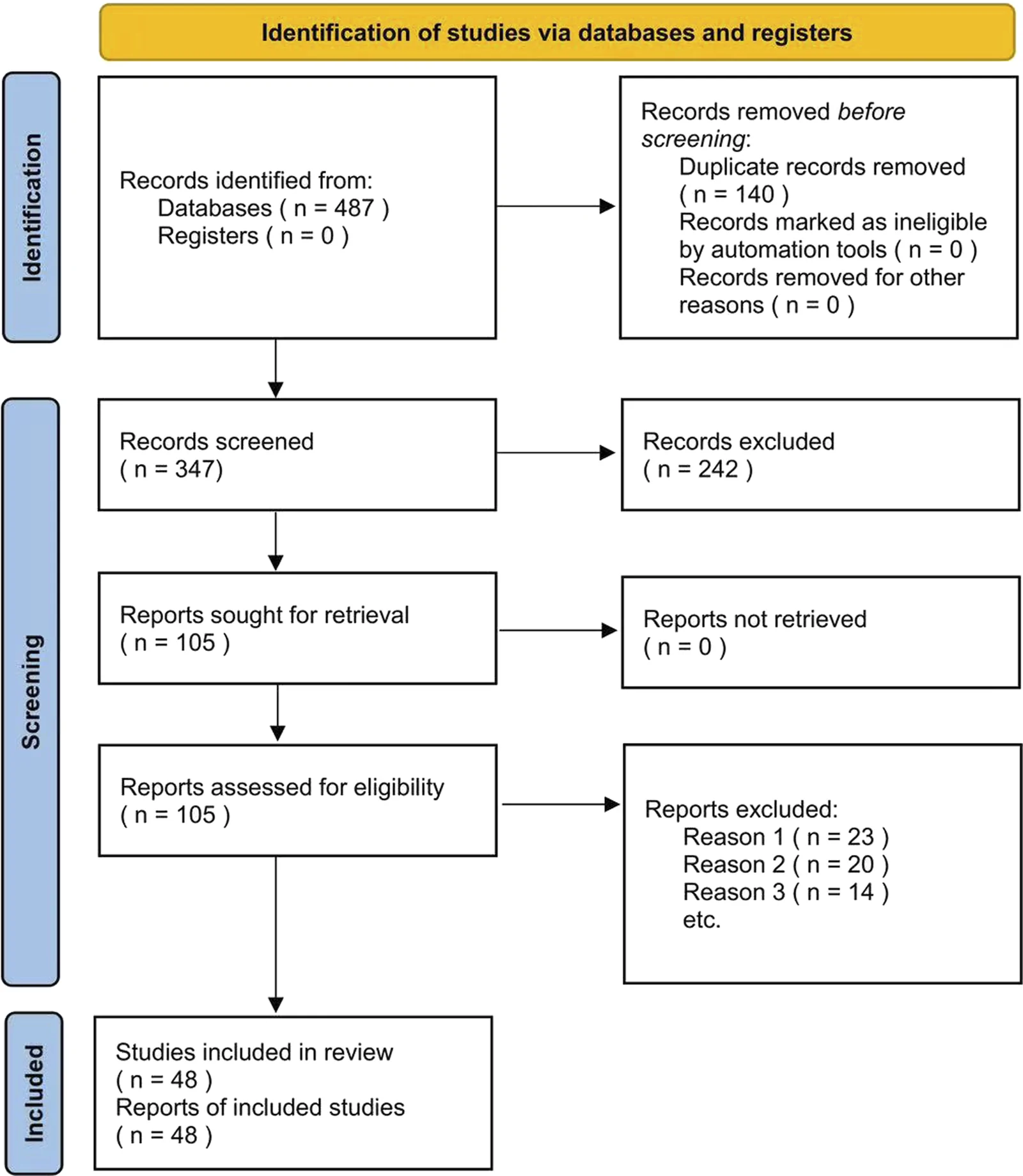
PRISMA flow diagram of the literature search and screening process. The diagram details the systematic identification, screening, eligibility assessment, and final inclusion of studies for this narrative review. A total of 487 records were identified through database searching. After removing duplicates, 347 records were screened based on titles and abstracts. Of these, 242 records were excluded as they were irrelevant to PAR1 or diabetic complications. Subsequently, 105 full-text articles were assessed for eligibility, among which 57 were excluded due to reasons such as lack of relevant mechanistic data, non-diabetic contexts, or being non-original reports. Finally, 48 studies were included in the narrative synthesis.

The literature screening was performed independently by two reviewers (Yanrui Wei and Bing Wang). Initially, titles and abstracts of all retrieved records were screened to remove clearly irrelevant articles. Subsequently, the full texts of the remaining articles were thoroughly assessed against the eligibility criteria. Disagreements during the selection process were resolved through discussion or, when necessary, by consultation with a third reviewer (Qiuyue Wang). The detailed screening process, including the number of records identified, excluded, and finally included, is illustrated in a PRISMA (Preferred Reporting Items for Systematic Reviews and Meta-Analyses) flow diagram (Figure 1). Relevant data were extracted from the included studies, focusing on: (1) PAR1 activation modes and downstream signaling cascades; (2) pathological roles in specific diabetic complications; and (3) pharmacological interventions targeting PAR1 (e.g., vorapaxar, 3K3A-APC, PZ-128). Given the narrative nature of this review, a formal quantitative meta-analysis was not performed; instead, the evidence was synthesized thematically to provide a comprehensive overview of the field.

## 3 Overview of PAR1: molecular structure, activation, signaling diversity and regulatory network

### 3.1 Structural features of PAR1

Protease-activated receptors (PARs) are cell-surface GPCRs with a typical seven-transmembrane helical structure composed of three intracellular loops and three extracellular loops. A conserved disulfide bond between extracellular loop 2 (ECL2) and the third transmembrane domain (TM3) is essential for maintaining receptor conformation. PAR1 activation depends on complex conformational rearrangements involving multiple extracellular loops, particularly ECL2 and ECL3. Among these loops, ECL2 is a critical determinant of ligand-recognition specificity and signaling efficacy. Compared with other PAR subtypes, PAR1 has a shallower, more surface-exposed ligand-binding pocket, a structural feature that may underlie its ability to mediate transient signaling (Ossovskaya and Bunnett, 2004; Han and Nieman, 2020). The N-terminus of PARs contains a signal peptide and a pro-domain, whereas the relatively short C-terminus harbors multiple post-translational modification sites, including N-glycosylation, ubiquitination, phosphorylation, and palmitoylation. To date, four PAR subtypes have been identified—PAR1, PAR2, PAR3, and PAR4—encoded by the F2R, F2RL1, F2RL2, and F2RL3 genes, respectively (Adams et al., 2011; Zhao et al., 2014). PARs are widely expressed in the cardiovascular, digestive, urinary, immune, and nervous systems and regulate important pathophysiological processes, including hemostasis, inflammation, pain transmission, cell proliferation, and tissue repair (Willis Fox and Preston, 2020; Ossovskaya and Bunnett, 2004). Unlike most GPCRs, PARs are not activated by soluble ligands. Instead, proteolytic cleavage of the extracellular N-terminus exposes a tethered ligand that binds intramolecularly to induce receptor conformational changes and initiate signal transduction (Ossovskaya and Bunnett, 2004).

### 3.2 Activation modes of PAR1

**FIGURE 2:**
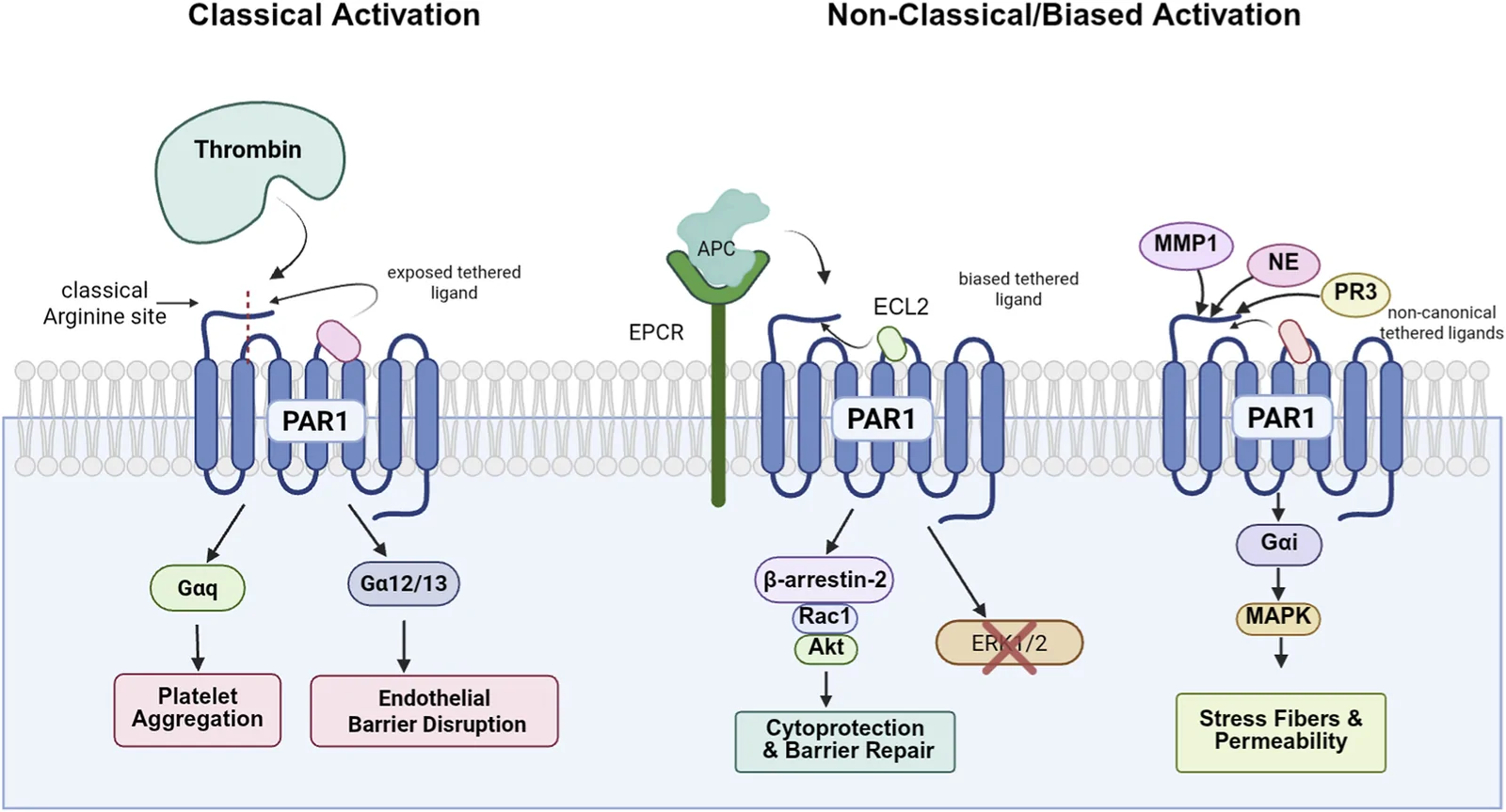
Mechanisms of PAR1 activation. PAR1 is activated by proteolytic cleavage of its extracellular N-terminus. Depending on the protease and cleavage site, activation can be divided into canonical (left) and non-canonical/biased (right) modes. Canonical cleavage by thrombin exposes the tethered ligand and activates Gαq- and Gα12/13-dependent signaling, promoting platelet aggregation and endothelial barrier disruption. In contrast, non-canonical cleavage by APC, MMP1, NE, or PR3 triggers biased signaling, including β-arrestin- or Gαi/MAPK-dependent pathways, leading to distinct cellular responses such as cytoprotection, barrier repair, or increased endothelial permeability.

PAR1 is activated by proteolytic cleavage of its extracellular N-terminus. Depending on the protease involved, the cleavage site, and the downstream signaling pathway, PAR1 activation can be classified as canonical or non-canonical, together contributing to its functional diversity (Figure 2). Proteases such as trypsin and thrombin cleave PARs at the canonical arginine site, exposing a tethered ligand that binds to the second extracellular loop of the receptor. This mode of activation typically engages multiple G-protein-dependent and β-arrestin-dependent signaling pathways (Zhao et al., 2014). Non-canonical activation has become a major focus of PAR1 research because it underlies biased signaling. Matrix metalloprotease-1 (MMP1), activated protein C (APC), neutrophil proteinase-3 (PR3), and neutrophil elastase (NE) cleave the PAR1 N-terminus at non-canonical sites, exposing tethered ligands that differ from the canonical sequence. These non-canonical ligands induce biased signaling, whereby different agonists cleaving PAR1 at distinct sites activate different downstream pathways and mediate distinct biological effects (Gieseler et al., 2013). For example, canonical PAR1 activation is mediated by thrombin cleavage at Arg-41, which triggers platelet activation and initiates pro-inflammatory G protein signaling in endothelial cells. In contrast, non-canonical cleavage by matrix metalloproteinase-1 (MMP-1) occurs at Asp-39 and preferentially couples PAR1 to Gα12/13 signaling, thereby promoting tumor cell invasion and increasing platelet responsiveness. MMP-13 cleaves PAR1 between Ser-42 and Phe-43 and may contribute to cardiac dysfunction through PAR1 activation in cardiac fibroblasts and cardiomyocytes. APC, a representative mediator of non-canonical PAR1 cleavage, can cleave PAR1 not only at the canonical Arg-41 site but also, more prominently, at Arg-46. Unlike thrombin-mediated cleavage, APC-mediated cleavage does not activate platelets but instead induces cytoprotective signaling, including anti-inflammatory, anti-apoptotic, and vascular barrier-protective effects (Willis Fox and Preston, 2020; Flaumenhaft and De Ceunynck, 2017).

In summary, site-specific proteolytic cleavage of PAR1 by different proteases enables the receptor to elicit divergent, and sometimes opposing, cellular responses within the same cell type or across different tissues. NNotably, this signaling bias depends not only on the cleavage site but also on the distinct modes of interaction among proteases, PAR1, and its coreceptors. For instance, thrombin efficiently binds to and directly cleaves PAR1, thereby engaging multiple downstream G protein pathways. In contrast, cytoprotective signaling induced by APC, factor VIIa (FVIIa), and factor Xa (FXa) largely depends on Gla domain-mediated engagement of coreceptors such as endothelial protein C receptor (EPCR) and integrin Mac-1. For example, APC binding to EPCR facilitates phosphorylation of the intracellular region of PAR1 by specific kinases, which induces β-arrestin 2 recruitment and subsequent activation of downstream cytoprotective signaling (Willis Fox and Preston, 2020; Flaumenhaft and De Ceunynck, 2017).

### 3.3 Core signaling pathways of PAR1

**FIGURE 3:**
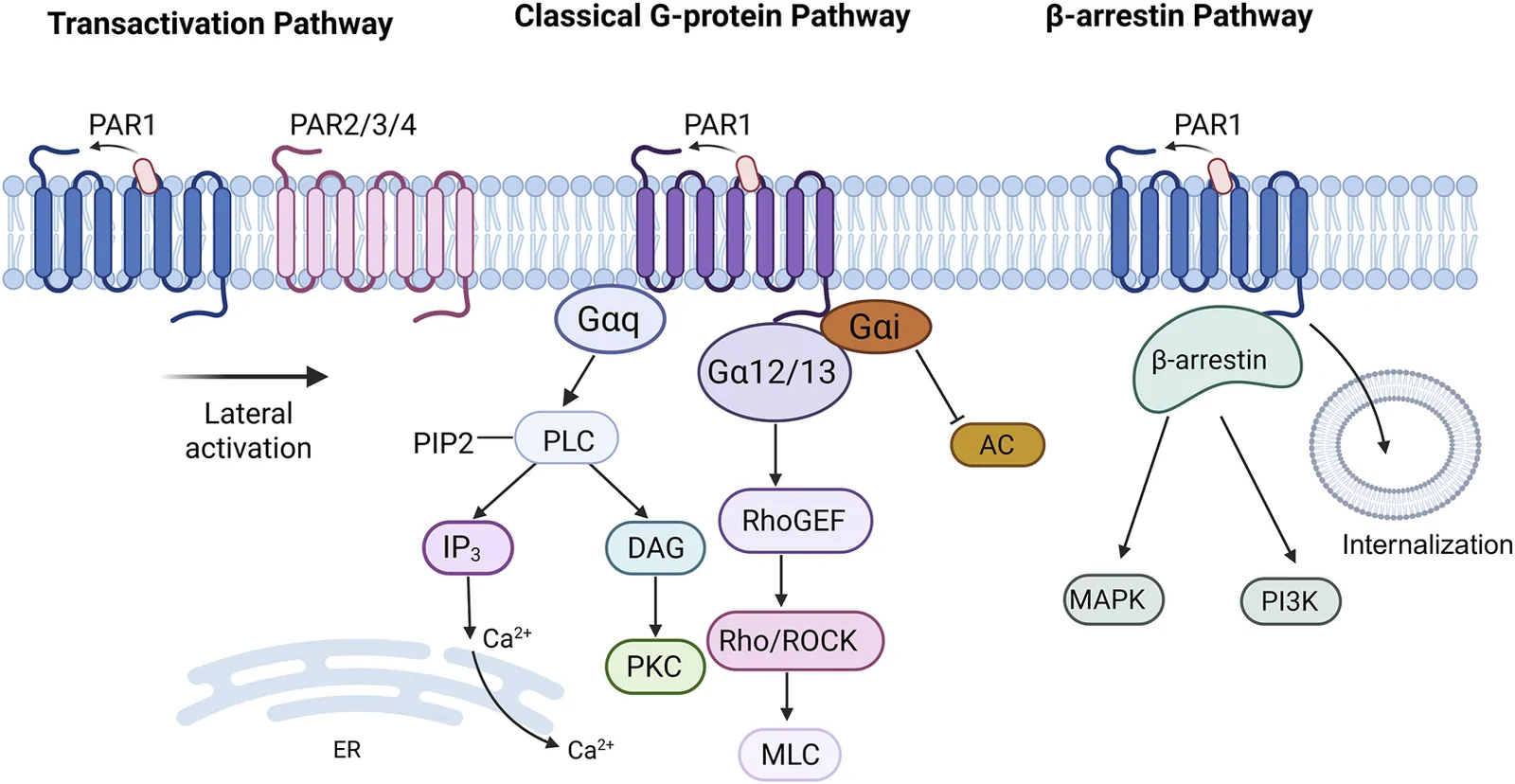
Core downstream signaling pathways and activation modes of PAR1. PAR1 signaling is initiated by proteolytic cleavage of its extracellular N-terminus and varies according to the protease involved and the cleavage site. As shown, PAR1 activation engages three major signaling modes: transactivation of neighboring PAR family receptors, canonical G-protein-dependent signaling through Gαq, Gα12/13, and Gαi, and β-arrestin-dependent G-protein-independent signaling. Together, these pathways mediate diverse effects including Ca2^+^ mobilization, PKC activation, cytoskeletal remodeling, MAPK signaling, receptor internalization, cytoprotection, and endothelial barrier regulation.

PAR signaling activates several major pathways (Figure 3). First, the canonical G-protein-dependent pathway, in which receptor activation signals through heterotrimeric guanine nucleotide-binding proteins (G proteins) and their downstream effectors. PARs can couple to the Gα subunits of various G proteins, including Gαi, Gα12/13, and Gαq, to activate downstream signaling messengers, thereby inducing numerous cellular and physiological effects. PAR1-mediated GDP-GTP exchange on Gαq activates phospholipase C, leading to PIP2 hydrolysis and generation of inositol triphosphate (IP3) and diacylglycerol (DAG), which mediate calcium release and protein kinase C activation, respectively. IP3 binds to its receptors to trigger intracellular calcium release and activation of downstream signaling proteins. PAR1-mediated activation of Gα12/13 stimulates RhoGEF, which in turn activates the Rho/ROCK pathway, resulting in increased myosin light chain phosphorylation. PAR1-mediated activation of Gαi inhibits adenylyl cyclase (Adams et al., 2011; Flaumenhaft and De Ceunynck, 2017).

Second, the β-arrestin-mediated, G-protein-independent pathway, in which β-arrestins form PAR–β-arrestin signaling scaffolds and mediate receptor internalization, thereby regulating effector molecules such as MAPK and PI3K and enabling ligand-specific signal transduction (Adams et al., 2011; Ma et al., 2025).

Third, PAR1 can also signal through receptor transactivation. After proteolytic activation, PAR1 can transactivate uncleaved PAR1, other PAR family members (e.g., PAR2, PAR3, and PAR4), and even receptors from other families, such as EGFR, through intermolecular signaling or direct physical interactions, without requiring additional cleavage. This mechanism complements classical intramolecular activation and further expands the signaling diversity of PAR1 (Gieseler et al., 2013).

### 3.4 Multidimensional pathophysiological regulatory network of PAR1

During the progression of vascular pathophysiology, the multidimensional functions of PAR1 are shaped by its structural conformational features and mechanisms of biased agonism. Subtle allosteric changes in the receptor-binding pocket allow different proteases to selectively stabilize distinct receptor conformations, thereby inducing divergent, and sometimes opposing, downstream biological effects through the same receptor (Ma et al., 2025; Onaran and Costa, 2021). This conformationally determined functional differentiation is further regulated in a spatiotemporal manner by endothelial mechanotransduction within the vascular microenvironment. By sensing dynamic changes in fluid shear stress, endothelial cells modulate their sensitivity to distinct chemical signals (Ossovskaya and Bunnett, 2004; Jackson et al., 2019). During severe infection or tissue injury, PAR1 serves as a central mediator of coagulation-immune crosstalk by linking tissue factor (TF) expression in innate immune cells to thrombin generation and by establishing a self-amplifying loop between pro-inflammatory cytokines and the coagulation cascade. Once this local interaction becomes dysregulated, it may progress to thromboinflammation, characterized by loss of endothelial antithrombotic and anti-inflammatory homeostasis. In this setting, PAR1 activation drives platelet activation and aggregation, induces neutrophil extracellular trap release, and facilitates widespread microthrombus formation in the systemic microvasculature, thereby contributing to multiple organ dysfunction syndrome (Jackson et al., 2019; Opal and Esmon, 2002). In chronic metabolic diseases, PAR1 axis-mediated vascular injury is further embedded in the persistent immunometabolic dysregulation of DM. Persistent hyperglycemia, lipotoxicity, and mitochondrial dysfunction trigger immunometabolic reprogramming and aberrant receptor glycosylation, leading to disruption of the cytoprotective PAR1 signaling axis and sustained pathological biased cleavage. These processes may ultimately accelerate renal podocyte apoptosis and fibrovascular degeneration (Ossovskaya and Bunnett, 2004; Narongkiatikhun et al., 2024).

In conclusion, PAR1 is not merely a conventional thrombin sensor; its role in vascular pathophysiology spans multiple dimensions, including coagulation, inflammation, immunity, and metabolism. These multidimensional regulatory mechanisms do not operate in isolation. Conformational bias determines the directionality of PAR1 signaling in response to different proteases, whereas endothelial mechanosensing and the metabolic microenvironment jointly shape the spatiotemporal specificity of its functional output. The flexibility and complexity of this multi-tiered regulation enable PAR1 to assume multiple pathological roles in chronic metabolic diseases and provide a mechanistic basis for its involvement in DM and its chronic complications.

## 4 Mechanisms of PAR1 in DM and its chronic complications

### 4.1 PAR1 in the development and progression of type 2 diabetes mellitus

Type 2 diabetes mellitus (T2DM) is characterized by hyperglycemia and is closely associated with chronic inflammation and coagulation abnormalities. Obesity-associated T2DM is accompanied by chronic low-grade inflammation, which induces tissue factor (TF) expression in monocytes and adipose tissue, thereby activating the extrinsic coagulation cascade and sustaining thrombin generation. TF signaling further contributes to adipose tissue inflammation, insulin resistance, and hepatic steatosis through PAR1 and PAR2 activation, highlighting a bidirectional pathological link between coagulation and metabolic dysfunction (Samad and Ruf, 2013).

Given the close interplay between coagulation and metabolic dysregulation, recent research has shifted from hypercoagulability per se toward the signaling mechanisms of PAR family receptors in insulin target organs. Among these receptors, PAR1, a high-affinity thrombin receptor, has drawn increasing attention. Because insulin resistance is a central feature of T2DM, whether PAR1 signaling directly contributes to its development has become an important research question. In the thrombin-rich microenvironment of T2DM, persistent activation of endothelial PAR1 upregulates protein tyrosine phosphatase 1B (PTP1B) through the Gαq/PLC-β/PKC pathway. PTP1B dephosphorylates tyrosine residues on the insulin receptor, thereby impairing insulin signaling (Rajala and Griffin, 2025). Reduced insulin receptor activity limits transendothelial insulin delivery and impairs NO-dependent vasodilation and tissue perfusion, indicating that reduced vascular reactivity in insulin-resistant states is closely associated with endothelial dysfunction (Tack et al., 1998). These effects exacerbate systemic insulin resistance and hyperglycemia. Endothelial insulin resistance reduces endothelial nitric oxide synthase (eNOS) activity and NO release, leading to capillary rarefaction and decreased blood flow to parenchymal tissues such as skeletal muscle. Impaired insulin and glucose delivery to peripheral tissues further aggravates systemic insulin resistance (Blackwood et al., 2024). Notably, endothelial PAR1 inhibition restores insulin receptor signaling and improves insulin transport, thereby enhancing systemic insulin sensitivity (Rajala and Griffin, 2025). Beyond the vascular endothelium, PAR1 is also expressed in tissues involved in glucose and lipid metabolism, including pancreatic islets, liver, and adipose tissue. These tissues regulate insulin secretion, hepatic glucose metabolism, adipokine release, and inflammatory signaling. Aberrant PAR1 activation in these compartments may contribute to systemic insulin resistance and glucose dysregulation through tissue-specific mechanisms.

PAR1 is expressed on pancreatic β-cells. Kreutter et al. showed that APC activates PI3K/Akt anti-apoptotic signaling through the EPCR/PAR1 pathway, thereby preserving β-cell function and restoring glucose-stimulated insulin secretion. However, whether persistent hyperthrombinemia in T2DM shifts PAR1 signaling toward pro-apoptotic effects remains unclear (Kreutter et al., 2017). These findings suggest that PAR1 function in β-cells is ligand- and microenvironment-dependent and may help explain the progressive decline in insulin secretory capacity in T2DM.

Hepatocytes also express PAR1. However, using hepatocyte-specific knockout models, Cline et al. showed that hepatocyte PAR1 does not directly drive high-fat diet-induced hepatic steatosis or insulin resistance. Instead, PAR1 activation at the canonical thrombin cleavage site R41 in non-parenchymal cells, such as macrophages and endothelial cells, may indirectly contribute to hepatic insulin resistance by driving systemic pro-inflammatory signaling. Consistently, the biased modulator NRD-21, which targets this site, reduces hepatic lipid accumulation and HOMA-IR while increasing active GLP-1 levels (Cline et al., 2024), supporting an indirect role for PAR1 in hepatic insulin resistance.

In adipose tissue, PAR1 and PAR4 participate in thrombin-driven inflammation and metabolic dysfunction. Strande and Phillips showed that human adipose tissue and adipocytes express PAR1, mainly in microvessels, and PAR4, mainly in adipocytes and preadipocytes. Thrombin stimulates adipocyte secretion of pro-inflammatory cytokines, including IL-1β, IL-6, TNF-α, and MCP-1, as well as VEGF, through PAR signaling (Strande and Phillips, 2009). *In vivo*, Kleeschulte et al. found that PAR4 is upregulated in visceral white adipose tissue under obese conditions. PAR4 knockout attenuates high-fat diet-induced adipocyte hypertrophy, macrophage infiltration, and glucose intolerance, suggesting that PAR4 may be the dominant thrombin-responsive receptor mediating adipose tissue inflammation and insulin resistance (Kleeschulte et al., 2024). Beyond white adipose tissue, brown adipose tissue (BAT) is also regulated by coagulation factor/PAR1 signaling. Hayashi et al. found that obesity upregulates tissue factor (TF), coagulation factor VII (FVII), activated factor X (FXa), and PAR1 in BAT. Through FXa/PAR1 signaling, endogenous and circulating coagulation factors induce mitochondrial calcium overload and excessive ROS production, leading to BAT whitening, impaired thermogenesis, and systemic glucose intolerance (Hayashi et al., 2022). Although PAR4 appears to be the principal thrombin-responsive receptor in white adipose tissue, PAR1 may have a distinct role in BAT thermogenic dysfunction and adipose tissue microvascular inflammation.

The pro-inflammatory effects of the thrombin/PAR signaling axis are not limited to metabolic organs. In atrial cardiomyocytes, Mittendorff et al. showed that obesity upregulates PAR1 and PAR4, and that PAR4 activation facilitates NLRP3 inflammasome-dependent IL-1β maturation and release while cross-activating c-Met signaling (Mittendorff et al., 2025). These findings suggest that PAR family receptors may link obesity-related cardiovascular inflammation with metabolic dysregulation.

In summary, thrombin and activated factor X (FXa) promote key pathological processes in cardiometabolic disease, including inflammation, insulin resistance, hepatic steatosis, and atherosclerosis, through PAR1 and PAR2 signaling in adipose tissue, liver, and vasculature. The differential expression of PAR1 and PAR2 in hematopoietic and non-hematopoietic cells contributes to their tissue-specific functions and supports the therapeutic rationale for targeting PAR signaling in T2DM and its complications (Hara et al., 2023). Collectively, these findings implicate PAR1, a high-affinity thrombin receptor, in the onset and progression of T2DM. Targeted modulation of PAR1 signaling may provide a novel strategy for preventing or treating T2DM and its chronic complications.

### 4.2 Roles of PAR1 in chronic complications of DM

In addition to its role in insulin resistance, PAR1 contributes to the initiation and progression of both microvascular and macrovascular diabetic complications. Hyperglycemia, oxidative stress, and inflammatory mediators upregulate PAR1 expression in the kidney, retina, peripheral nerves, and cardiovascular system, thereby promoting target-organ damage through shared pro-inflammatory, pro-fibrotic, and pro-angiogenic mechanisms and forming an interconnected signaling network (Table 1). The roles of PAR1 in diabetic kidney disease, diabetic retinopathy, diabetic peripheral neuropathy, and diabetic cardiovascular disease are summarized below.

**TABLE 1:** Major mechanisms of PAR1 in chronic diabetic complications.

Complication	Activating factors	PAR1 localization	Core signaling pathways	Pathological effects
DKD	High-glucose; thrombin; FXa; urokinase; TF-mediated coagulation cascade	Glomerular endothelial cells; mesangial cells; podocytes; tubular epithelial cells; renal fibroblasts	① Pro-inflammatory: High glucose → PAR1 activation → NF-κB phosphorylation/NLRP3 inflammasome activation → Caspase-1 activation → IL-1β and IL-18 release ② Pro-fibrotic and cellular injury: 1) Renal tubular epithelial cells: High glucose → transcription factor Egr1 activation → upregulation and activation of TGF-β1/Smad3 pathway 2) Acute-to-chronic kidney injury transition: FXa → PAR1 → Gαi/Ca2+ → PDGF secretion → ECM remodeling and deposition 3) Podocyte injury: Serine proteases → PAR1 → PLC-γ1 → TRPC6 channel opening → sustained intracellular Ca2+ overload → p38 MAPK pathway activation → podocyte foot process retraction and cytoskeletal rearrangement	Cytokine release; coagulation-inflammation amplification; mesangial expansion; ECM deposition; tubulointerstitial fibrosis; podocyte apoptosis; glomerulosclerosis
DR	Thrombin, MMP-1, IL-1β, TNF-α	Retinal vascular endothelial cells; CD45^+^ leukocytes; myofibroblasts; Müller cells	① Pathological angiogenesis/fibrosis axis: Thrombin/MMP-1 → cleavage of PAR1 extracellular N-terminus → induction of VEGF overexpression and enhanced migration in vascular endothelial cells ② Müller cell fibrotic pathway: Thrombin → PAR1 → atypical PKC-ζ → sustained Raf/MEK/ERK phosphorylation → pathological glial proliferation of Müller cells ③ Early neurodegeneration: Early diabetes → local thrombin overactivation → prominent PAR1 nuclear translocation (enrichment in nuclei of ganglion cell layer, etc.) → regulation of intranuclear transcription	Early: neurosensory retinal dysfunction (decreased scotopic and photopic ERG amplitude ratios) Late:pathological angiogenesis, fibrovascular membrane contraction, retinal scarring, and progression to proliferative DR (PDR)
DPN	High glucose; thrombin with concentration-dependent effects	Schwann cells; small nerve fibers	① Glucotoxicity–protease pathogenic crosstalk: Systemic metabolic disturbances (polyol pathway activation) → ROS burst in Schwann cells, impaired mitochondrial respiratory chain complex activity → collapse of mitochondrial membrane potential; toxic crosstalk with high-concentration thrombin-mediated PAR1 overactivation ② Endogenous neuroprotective pathway: Physiological/low-concentration thrombin → EPCR pathway activation in Schwann cells → biased APC/PAR1 activation → preservation of blood–nerve barrier integrity and promotion of nerve repair	Nodal disruption; schwann cell demyelination; axonal degeneration; small nerve fiber loss; reduced nerve conduction velocity
DCM and ASCVD	DCM: High glucose; KLK8 ASCVD: High glucose; PMPs; CAPN1	Cardiac fibroblasts; myocardial endothelial cells; vascular endothelial cells; monocytes/macrophages	① DCM myocardial fibrosis pathway: High glucose → upregulation of KLK8 → direct cleavage and activation of fibroblast PAR1 → cross-activation of TGF-β1/Smad3 cascade → driving cardiac fibroblast-to-myofibroblast transition and collagen remodeling ② ASCVD endothelial injury and vascular inflammation pathway: High glucose → platelet release of CAPN1-rich microparticles → direct cleavage of PAR1 N-terminal domain → Gα-dependent pathway → PKCα/β and ERK1/2 phosphorylation → activation of TACE → pathological EPCR shedding and massive TNF-α release ③ Vascular endothelial permeability and polarization axis: 1) Activation of RhoA/NF-κB pathway → increased vascular permeability; 2) Tissue factor cytoplasmic domain → upregulation of PAR1-Rac1 axis → driving cardiac/vascular resident macrophage polarization toward pro-inflammatory M1 phenotype	DCM: EndMT; myocardial fibrosis; interstitial and cardiac remodeling; electrophysiological disturbances ASCVD: Vascular inflammation; endothelial dysfunction; monocyte adhesion; plaque vulnerability

#### 4.2.1 PAR1 and diabetic kidney disease

Diabetic kidney disease (DKD) arises from the interplay of multiple signaling pathways, among which chronic inflammation and fibrosis are central and mutually reinforcing processes. Upon activation, PAR1 mediates inflammatory and fibrotic responses through multiple downstream pathways, suggesting that it may contribute to the initiation and progression of DKD. In the kidney, PARs are mainly expressed in renal epithelial cells, endothelial cells, and podocytes, where they regulate glomerular function, microvascular homeostasis, and inflammatory responses under both physiological and pathological conditions (Bagang et al., 2023). Under pathological conditions, PARs contribute to kidney injury by modulating inflammation, hemodynamics, electrolyte balance, and podocyte integrity, with PAR1 and PAR2 being the predominant subtypes, particularly in the regulation of vascular tone (Palygin et al., 2016). Among PAR subtypes, PAR1 is highly expressed in renal tissue and has been implicated in various kidney diseases. Serine proteases, including thrombin, plasmin, and kallikrein, can activate PARs and thereby modulate renal function (Fedoriuk et al., 2025).

High glucose is a major trigger of PAR1 activation and upregulation in the kidney. Multiple studies have shown that high glucose markedly increases PAR1 mRNA and protein expression in the renal cortex, glomerular mesangial cells, podocytes, and renal tubular epithelial cells in diabetic models. PAR1 activation mediates inflammation mainly through two pathways. First, it activates NF-κB signaling, increases p65 phosphorylation, and enhances the transcription and release of pro-inflammatory cytokines. Second, it activates the nucleotide-binding oligomerization domain-like receptor protein 3 (NLRP3) inflammasome, increases cleaved caspase-1 expression, and promotes the secretion of IL-1β and IL-18, thereby amplifying the inflammatory response. As a major thrombin receptor, PAR1 upregulation may also enhance coagulation activation and thrombogenicity in DKD, creating a vicious coagulation–inflammation cycle that aggravates renal injury (Tang et al., 2020).

In addition to promoting inflammatory cascades, another key pathogenic effect of PAR1 in DKD progression is driving renal fibrosis and cellular injury, an effect that displays marked cell-type specificity and signaling pathway diversity. Blanc-Brude et al. showed that coagulation factor Xa directly cleaves and activates PAR1, triggering Gαi-dependent intracellular Ca^2+^ signaling, upregulating pro-collagen α1(I) transcription, and promoting collagen synthesis. PAR1 activation also stimulates fibroblast proliferation and collagen deposition, partly through an autocrine platelet-derived growth factor (PDGF)-dependent mechanism (Blanc-Brude et al., 2005). Xu et al. identified a thrombin-independent profibrotic mechanism in a DKD model. High glucose activates early growth response factor 1 (Egr1) in renal tubular epithelial cells, enhances its binding to the PAR1 promoter, and transcriptionally upregulates PAR1. This Egr1/PAR1/TGF-β1 axis enhances TGF-β1/Smad signaling, tubulointerstitial fibrosis, and extracellular matrix deposition, and appears to be insensitive to thrombin inhibition (Xu et al., 2023). This study offers a distinct perspective, demonstrating that high glucose induces the transcription factor Egr1 to directly bind to the PAR1 promoter, thereby transcriptionally upregulating PAR1 expression and activating the TGF-β1/Smad pathway, a process that is insensitive to thrombin inhibition. This thrombin-independent mechanism may help explain why anticoagulation therapy alone is insufficient to delay renal fibrosis in DKD, although it should be reconciled with previous thrombin-centered models. However, given the complexity of the *in vivo* microenvironment, future studies are needed to elucidate whether other endogenous proteases mediate cross-activation and synergistic regulation of highly expressed PAR1. PAR1-driven fibrosis may also contribute to the transition from acute to chronic kidney injury by promoting ERK-MAPK-dependent activation of TGF-β/Smad3 signaling and extracellular matrix accumulation (Lok et al., 2023). In glomeruli, PAR1-mediated podocyte injury represents another important mechanism of DKD progression. Bohovyk et al. showed that, under diabetic conditions, elevated serine proteases such as thrombin and urokinase activate podocyte PAR1, which upregulates and opens transient receptor potential canonical 6 (TRPC6) channels through G protein-coupled pathways, including PLC-γ1. The resulting sustained intracellular Ca^2+^ overload induces lamellipodial retraction, cytoskeletal remodeling, proteinuria, p38 MAPK activation, apoptosis, and ultimately glomerulosclerosis (Bohovyk et al., 2023).

Beyond cellular injury and fibrosis, PAR1 may also link coagulation, inflammation, and microvascular dysfunction in DKD. The apelinergic system, VEGF/VEGFR axis, and NO/NOS pathway exert stage-dependent effects in DKD, being renoprotective in early disease but contributing to inflammation, fibrosis, and microvascular injury when dysregulated (Gaydarski et al., 2024a). In hypertensive renal injury models, renal damage is accompanied by capillary rarefaction, VEGF depletion, compensatory APLNR and nNOS upregulation, and impaired endothelial NO-dependent vasodilation and angiogenesis (Gaydarski et al., 2024b). Similar maladaptive microvascular responses may occur in DKD. Because PAR1 activation can modulate endothelial survival and angiogenic signaling, aberrant PAR1 activity may interact with the VEGF/APLNR axis and contribute to capillary rarefaction in DKD. Future studies should determine whether PAR1 antagonism can preserve capillary density by modulating these compensatory vascular pathways.

PAR1-mediated activation of the NLRP3 inflammasome may amplify inflammation and interact with emerging injury mechanisms, including pyroptosis, ferroptosis, and dysregulated autophagy, thereby contributing to progressive DKD injury (Liu et al., 2021; Wu and Chen, 2022; Zhang et al., 2021). Although direct evidence in DKD remains limited, studies in other disease contexts suggest that these pathways warrant further investigation. A study on allergic airway disease revealed that allergens, acting through PAR1 on lung epithelial cells, trigger ferritinophagy and expand the intracellular labile iron pool. The iron chaperone PCBP2 delivers iron directly to GSDMD and catalyzes a local Fenton reaction, generating active N-terminal GSDMD fragments in a protease-independent manner, resulting in pore formation and IL-33 release (Chen et al., 2026). This finding delineates a complete signaling cascade in which PAR1-triggered ferritinophagy leads to iron-dependent oxidative cleavage of GSDMD, providing direct mechanistic evidence for PAR1-mediated crosstalk between ferroptosis and non-canonical pyroptosis pathways. Furthermore, in a model of neuroinflammatory injury following ischemic stroke (IS), Luo et al. reported that PAR1 expression was upregulated and that PAR1 inhibited autophagy by suppressing ULK1 complex formation and reducing LC3-II levels through the PI3K/AKT/mTOR pathway (Luo et al., 2023). These observations suggest that PAR1 may also contribute to inflammation, cell death, and impaired autophagy in DKD renal cells, although this possibility requires direct experimental validation.

In summary, PAR1 promotes DKD progression by enhancing inflammatory signaling, fibrotic responses, podocyte injury, and glomerulosclerosis. These findings support PAR1 and its downstream pathways, including TRPC6, TGF-β1/Smad, and Ca^2+^ signaling, as potential therapeutic targets in DKD. Future studies should use more physiologically relevant models, such as multicellular co-culture systems and organ-on-a-chip platforms, together with toxicological evaluation and formulation optimization, to support the clinical translation of PAR1-targeted therapies.

#### 4.2.2 PAR1 and diabetic retinopathy

Diabetic retinopathy (DR), a common microvascular complication of DM, is classified into non-proliferative and proliferative stages. It is primarily driven by chronic hyperglycemia-induced retinal vascular injury and may lead to visual impairment or blindness. Advanced DR is characterized by pathological retinal neovascularization, which can result in vitreous hemorrhage, retinal detachment, and neovascular glaucoma (Hu et al., 2024; Xu et al., 2025). PAR1 is an important regulator of this pathological angiogenic process.

PAR1 is highly expressed in the fibrovascular membranes of proliferative diabetic retinopathy (PDR), particularly in vascular endothelial cells, CD45-positive leukocytes, and myofibroblasts, which are involved in angiogenesis and inflammation. Its activation is driven by pro-inflammatory cytokines such as IL-1β and TNF-α and may also be directly promoted by the diabetic microenvironment. In addition, thrombin and MMP-1 levels are markedly increased in the vitreous and retinal tissues of patients with DM. Both proteases activate PAR1 by cleaving its extracellular N-terminus and exposing the tethered ligand, thereby stimulating VEGF secretion and endothelial cell migration and ultimately promoting pathological angiogenesis (Abu El-Asrar et al., 2016). Moreover, in diabetic retinopathy (DR), Müller cell dysfunction contributes to inflammation, neuronal injury, and pathological angiogenesis. Müller cells are the predominant retinal glial cells and span the entire retinal thickness. In DR, they undergo functional and phenotypic changes, including gliosis, VEGF overexpression, ion and water channel dysregulation, oxidative stress, and inflammation. Although reactive gliosis may initially be protective, persistent Müller cell dysfunction can accelerate disease progression (Bringmann et al., 2006). Lee-Rivera et al. showed that human retinal Müller cells highly express PAR1. Upon thrombin stimulation, PAR1 activates the atypical protein kinase PKC-ζ rather than the canonical Ras or PI3K pathways, thereby regulating the Raf/MEK/ERK cascade and inducing sustained ERK1/2 phosphorylation. This pathway facilitates pathological Müller cell proliferation and may contribute to contractile fibroglial membrane formation, retinal scarring, and progression of proliferative diabetic retinopathy (Lee-Rivera et al., 2016).

The pro-angiogenic and fibroproliferative roles of PAR1 are mainly observed in mid-to-late DR. However, DR is now recognized as a neurovascular disease, in which retinal neurodegeneration may precede overt microvascular lesions. PAR1 signaling may also participate in this early neuronal injury phase. Goldberg et al. showed that local retinal thrombin/PAR1 signaling contributes to early retinal injury. In an early-stage streptozotocin-induced diabetic mouse model, retinal thrombin activity and PAR1 mRNA and protein levels were increased. The study also reported PAR1 nuclear translocation in the ganglion cell layer, inner nuclear layer, and outer nuclear layer, suggesting a possible role in nuclear transcriptional or chromatin-associated regulation. Before overt disruption of the retinal vascular barrier or gross retinal morphology, pathway activation was already associated with electrophysiological dysfunction, including reduced scotopic a- and b-wave and photopic b-wave amplitudes on electroretinography. Topical PARIN5, a thrombin-PAR1 modulator, inhibited thrombin activity, reduced PAR1 nuclear accumulation, and improved retinal electrophysiological function. These findings suggest that targeting retinal thrombin/PAR1 signaling may protect retinal neurons before overt vascular lesions develop. However, the study was limited by its reliance on an animal model, analysis at a single early time point, and relatively small sample size. Further studies are needed to clarify the mechanisms of PAR1 nuclear translocation and evaluate the ocular pharmacokinetics and long-term safety of PARIN5 (Goldberg et al., 2026).

In summary, PAR1-mediated signaling may participate in multiple stages of DR. In early disease, local thrombin/PAR1 activation and PAR1 nuclear translocation are associated with neurosensory retinal dysfunction. As DR progresses, PAR1 activation through inflammatory and coagulation-related pathways may increase VEGF expression, endothelial migration, pathological angiogenesis, and fibroproliferation, thereby contributing to PDR. Thus, targeting the PAR1 signaling axis may provide a therapeutic strategy for both early neuroprotection and late-stage vascular intervention in DR.

#### 4.2.3 PAR1 and diabetic peripheral neuropathy

Diabetic peripheral neuropathy (DPN) is a common chronic complication of DM, typically characterized by pain and numbness in the limbs, especially the lower extremities and feet. Because DPN involves multiple sites in the nervous system, its clinical manifestations are heterogeneous (Pop-Busui et al., 2017). The pathogenesis of DPN is closely associated with chronic hyperglycemia-induced inflammation, oxidative stress, and mitochondrial dysfunction. These processes interact to promote structural nerve fiber damage, including axonal degeneration and segmental demyelination (Román-Pintos et al., 2016). Schwann cells, the principal glial cells of the peripheral nervous system, are responsible for myelin sheath formation. As DPN is closely associated with demyelination and Schwann cell apoptosis, Schwann cell dysfunction is considered a major contributor to its pathogenesis (Shavit-Stein et al., 2020).

PAR1 is expressed in both the peripheral and central nervous systems. In the peripheral nervous system, it is localized to Schwann cell microvilli at the nodes of Ranvier and to neuromuscular junctions, whereas in the central nervous system it is found on the perisynaptic end-feet of astrocytes. Through its interaction with thrombin, PAR1 exerts both neuroprotective and neurotoxic effects in a concentration-dependent manner: high thrombin concentrations disrupt nerve conduction, whereas low concentrations may exert neuroprotective effects via endothelial protein C receptor signaling in Schwann cells (Gofrit and Shavit-Stein, 2019). In addition, APC signaling through PAR1 can reduce nerve injury and improve nerve function through anti-inflammatory and anti-apoptotic effects, preservation of blood-brain barrier integrity, and promotion of neural repair (Guo et al., 2013). In the diabetic microenvironment, hyperglycemia-driven metabolic disturbances increase local thrombin activity in neural tissues and activate the polyol and hexosamine pathways, thereby promoting oxidative stress and mitochondrial dysfunction. This glucotoxic environment induces reactive oxygen species (ROS) production in Schwann cells, impairs mitochondrial respiratory chain complexes I and IV, promotes lipid peroxidation, and disrupts mitochondrial membrane potential. These changes may interact with excessive PAR1 activation to amplify neural injury (Román-Pintos et al., 2016; Pompili et al., 2021). Diabetic vasculopathy may further disrupt the blood-nerve barrier, facilitate thrombin infiltration into nerve tissue, and aggravate nerve injury. In a streptozotocin (STZ)-induced diabetic neuropathy rat model, Shavit-Stein et al. showed that excessive thrombin pathway activation is associated with DPN. Diabetic rats exhibited increased thrombin activity in the sciatic nerve, disrupted nodes of Ranvier, and altered PAR1 immunoreactivity. Treatment with the thrombin inhibitor NAPAP or the serine protease inhibitor TLCK preserved nerve conduction velocity, reduced thermal latency, and maintained nodal integrity (Shavit-Stein et al., 2019). The authors further developed PARIN5, a pentapeptide modulator derived from the thrombin recognition site of PAR1, to selectively block thrombin-mediated PAR1 activation. In an STZ-induced DPN mouse model, PARIN5 preserved nodal integrity, restored small nerve fiber density, and improved nerve conduction and sensorimotor function without affecting systemic coagulation, supporting a pathogenic role of aberrant thrombin/PAR1 signaling in DPN. Selective inhibition of pathological PAR1 activation may thus provide neuroprotection while minimizing anticoagulation-related adverse effects, representing a promising etiological strategy for DPN (Shavit-Stein et al., 2020).

#### 4.2.4 PAR1 and diabetic cardiovascular disease

Diabetic cardiovascular disease mainly includes diabetic cardiomyopathy (DCM) and atherosclerotic cardiovascular disease (ASCVD). DCM is driven by cardiac microvascular dysfunction and myocardial metabolic disturbances, which induce oxidative stress and inflammatory signaling, leading to myocardial injury and pathological remodeling. Clinically, DCM is characterized by myocardial hypertrophy, fibrosis, and systolic and diastolic dysfunction and is a major cause of morbidity and mortality in patients with DM. Suppression of myocardial fibrosis is thus a central therapeutic strategy in DCM (Tan et al., 2020; Zhang M. et al., 2024). In diabetic cardiomyopathy (DCM), PAR1 may contribute to disease progression by acting on cardiac fibroblasts and endothelial cells. Using endothelium-specific PAR1 and PAR4 knockout models, Rajala and Griffin showed that loss of endothelial PAR signaling attenuates hyperglycemia-induced endothelial dysfunction (Rajala and Griffin, 2025). Under high-glucose conditions, kallikrein-related peptidase 8 (KLK8) is upregulated and proteolytically activates PAR1, promoting cardiac fibroblast-to-myofibroblast transition, collagen synthesis, and cell migration. PAR1 also activates TGF-β1/Smad3 signaling, which drives extracellular matrix remodeling and exacerbates myocardial fibrosis. Pharmacological PAR1 blockade with agents such as GAS or SCH79797 attenuates myocardial fibrosis, suggesting a potential therapeutic strategy for DCM (Zhang M. et al., 2024). Notably, the profibrotic activity of PAR1 is not its only biological function; PAR1 effects depend on cell type and receptor activation state. Gong et al. reported that, in the unactivated state, PAR1 can act as a scaffold for TGFβRII and inhibit TGF-β signaling, thereby promoting endothelial differentiation of embryonic stem cells (Gong et al., 2016). These findings suggest that PAR1-targeted therapies for diabetic cardiovascular complications should account for context-dependent and potentially divergent PAR1 functions.

ASCVD encompasses clinical conditions caused by atherosclerotic lesions, including coronary heart disease, ischemic stroke/transient ischemic attack, and peripheral arterial disease, and is a leading cause of disability and death in patients with DM. Its development involves multiple pathological mechanisms, including dyslipidemia, hypercoagulability, oxidative stress, accumulation of advanced glycation end products, and chronic inflammation (Jialal and Chaudhuri, 2019). In the vascular pathology of ASCVD, PAR1 may also contribute to vascular inflammation and endothelial dysfunction. Under hyperglycemic conditions, aberrantly activated platelets release platelet-derived microparticles (PMPs) enriched in active calpain-1 (CAPN1), which directly cleaves and activates the N-terminus of PAR1. Activated PAR1 then induces PKCα/β and ERK1/2 phosphorylation through a Gαq-dependent pathway, leading to activation of tumor necrosis factor-α-converting enzyme (TACE), EPCR shedding, and TNF-α release. PAR1 also activates the RhoA/NF-κB pathway, increasing endothelial permeability and p65 NF-κB phosphorylation. TNF-α upregulates intracellular adhesion molecule-1 (ICAM-1) and promotes monocyte adhesion, whereas EPCR shedding impairs APC-mediated cytoprotection, ultimately leading to vascular inflammation and endothelial dysfunction. These findings identify the CAPN1–PAR1 axis as a potential therapeutic target in diabetic cardiovascular and microvascular complications (Kyselova et al., 2020).

Given the established PAR1-driven mechanisms in DCM and ASCVD described above, the potential involvement of PAR1 in broader vascular pathological processes warrants further exploration. Endothelial dysfunction, endothelial-to-mesenchymal transition (EndMT), and macrophage polarization imbalance are key contributors to diabetic cardiovascular disease. Hyperglycemia induces endothelial inflammation, oxidative stress, and mitochondrial metabolic dysfunction, leading to endothelial injury and impaired microvascular homeostasis. It can also activate TGF-β and Wnt signaling to initiate EndMT, thereby promoting myocardial fibrosis, conduction abnormalities, and arrhythmias. In parallel, hyperglycemia favors pro-inflammatory M1 macrophage polarization and suppresses reparative M2 responses, sustaining myocardial inflammation, oxidative injury, and interstitial remodeling (Zhang S. et al., 2024; Wang et al., 2023; Yang et al., 2024). Although direct evidence linking PAR1 to hyperglycemia-induced inflammation, macrophage phenotypic switching, and vascular calcification in diabetic cardiovascular complications remains limited, findings from related models suggest that PAR1 may contribute to diabetic cardiovascular injury through pathway crosstalk. As mentioned earlier, obesity upregulates PAR1 and PAR4 expression in atrial cardiomyocytes, and PAR4 activation facilitates IL-1β maturation and release via an NLRP3 inflammasome-dependent pathway (Mittendorff et al., 2025), suggesting that in the diabetic myocardial microenvironment, PAR1 may similarly drive sterile inflammation through the NLRP3 inflammasome pathway. In a myocardial infarction model, Chong et al. found that the tissue factor cytoplasmic domain drives cardiac macrophage polarization toward a pro-inflammatory M1 phenotype by upregulating the PAR1-Rac1 axis, maintaining an elevated M1/M2 ratio and exacerbating cardiac inflammation and injury (Chong et al., 2021). Although this study was conducted in an ischemic rather than diabetic model, it supports the possibility that PAR1-related signaling may regulate cardiac macrophage polarization in diabetic cardiomyopathy. This hypothesis requires direct validation in diabetic models. In valve interstitial cells from aortic stenosis, TNF-α upregulates PAR1/2 expression, whereas PAR1 inhibition is associated with reduced expression of pro-calcific markers such as osteopontin and osteocalcin (Wypasek et al., 2020). These findings suggest that PAR1 may modulate osteogenic-like phenotypes in valve interstitial cells, although its causal role in vascular calcification requires further validation. In addition to platelet-derived CAPN1, matrix metalloproteinase 1 (MMP1) in the atherosclerotic plaque microenvironment can activate PAR1 at a non-canonical site. This activation promotes vascular inflammation and monocyte infiltration through the p38/NF-κB pathway, thereby contributing to atherosclerosis progression (Rana et al., 2018). Unlike CAPN1-mediated activation, the MMP1-PAR1 axis is not strictly dependent on hyperglycemia. This suggests that persistent PAR1 activation in DM-associated ASCVD may be maintained by multiple microenvironmental proteases rather than solely by hyperglycemia-induced platelet pathways. Taken together, these findings support the hypothesis that aberrant PAR1 activation may contribute to EndMT, pro-inflammatory macrophage polarization, myocardial interstitial remodeling, and fibrosis in diabetic cardiomyopathy. In ASCVD, PAR1 activation may impair endothelial barrier function, increase vascular permeability, and contribute to plaque inflammation, calcification, and instability. Future studies should directly validate the role of PAR1 in diabetic cardiovascular disease and evaluate its therapeutic potential.

In summary, PAR1 contribute to myocardial fibrosis and vascular inflammation through the KLK8/TGF-β1 and CAPN1/TACE/NF-κB axes, respectively, and thereby contributes to the development of DCM and ASCVD. These findings further support PAR1 as a potential therapeutic target in diabetic cardiovascular complications.

#### 4.2.5 Current challenges and knowledge gaps

Although existing studies implicate PAR1 in diabetic complications across multiple organs and cell types, several important limitations remain.

First, current model systems have limited pathophysiological relevance. Most animal studies rely on streptozotocin (STZ)-induced type 1 diabetes models or genetically modified mice, whereas longitudinal studies in composite T2DM models that better reflect clinical disease remain limited. Although conditional knockout approaches can overcome the embryonic lethality of global PAR1 deletion, spatiotemporally controlled PAR1 manipulation in specific cell types and organs, such as the kidney and retina, has not been systematically applied. A further challenge is the substantial species-specific divergence in PAR1 expression and distribution between humans and rodents. For instance, human platelets co-express PAR1 and PAR4, whereas mouse platelets lack PAR1 and instead express PAR3 and PAR4 (Ossovskaya and Bunnett, 2004). This interspecies disparity limits the direct extrapolation of antithrombotic and bleeding phenotypes from animal models to clinical settings. *In vitro*, the commonly used 30 mM high-glucose condition may induce nonspecific cellular stress. Moreover, most studies use static monolayer cultures that fail to capture multicellular interactions and dynamic physical cues, such as hemodynamic shear stress, present in native tissues.

Second, mechanistic studies remain insufficiently integrated and specific. Although multiple studies have characterized PAR1-mediated downstream pathways, several aspects remain incompletely elucidated, including the crosstalk among these pathways, the key transcription factors regulating PAR1 expression, the potential non-canonical nuclear functions of PAR1, such as the nuclear translocation observed in diabetic retinopathy (DR), and tissue-specific PAR1 signaling across organs. Existing studies also often examine PAR1 in isolation, with limited attention to the synergistic or antagonistic roles of other PAR family members, such as PAR2 and PAR4. In addition, the contribution of thrombin-independent activators, including granzymes and urokinase, has not been adequately distinguished.

Third, *in vivo* functional validation and clinical correlation remain insufficient. Some conclusions rely heavily on *in vitro* knockdown or pharmacological inhibition and lack rescue validation using tissue-specific gene deletion *in vivo*. Human studies are often limited by small sample sizes, single-complication cohorts, and insufficient clinical association data linking PAR1 expression or activation to disease severity. These limitations constrain the translation of experimental findings to human diabetes.

Finally, clinical translation faces several barriers. Current studies often focus on phenotypic interventions using natural products, such as sarsasapogenin, or nonspecific inhibitors, but the oral bioavailability, metabolic stability, and long-term safety of these agents remain insufficiently evaluated. In addition, most experimental systems do not fully reproduce clinically relevant pathological complexity, including the effects of comorbid hypertension and hyperlipidemia on PAR1 signaling. Most studies still examine individual complications in isolation and rarely assess PAR1 signaling in the context of coexisting DKD, DR, DPN, and cardiovascular disease. This limits understanding of the pathogenic contribution of PAR1 in patients with multiple diabetic complications. Preclinical data are also lacking on the combined use of PAR1 antagonists with standard therapies, such as ACEIs/ARBs and SGLT2 inhibitors.

Future studies should use tissue-specific conditional knockout models, multi-complication animal models, organoids, and multi-omics approaches to clarify PAR1-centered mechanisms. Organ-targeted delivery systems are also needed to facilitate the clinical translation of PAR1-targeted therapies, enable combination with current standards of care, and minimize systemic coagulation risks.

## 5 Drug development and clinical application targeting PAR1

**FIGURE 4:**
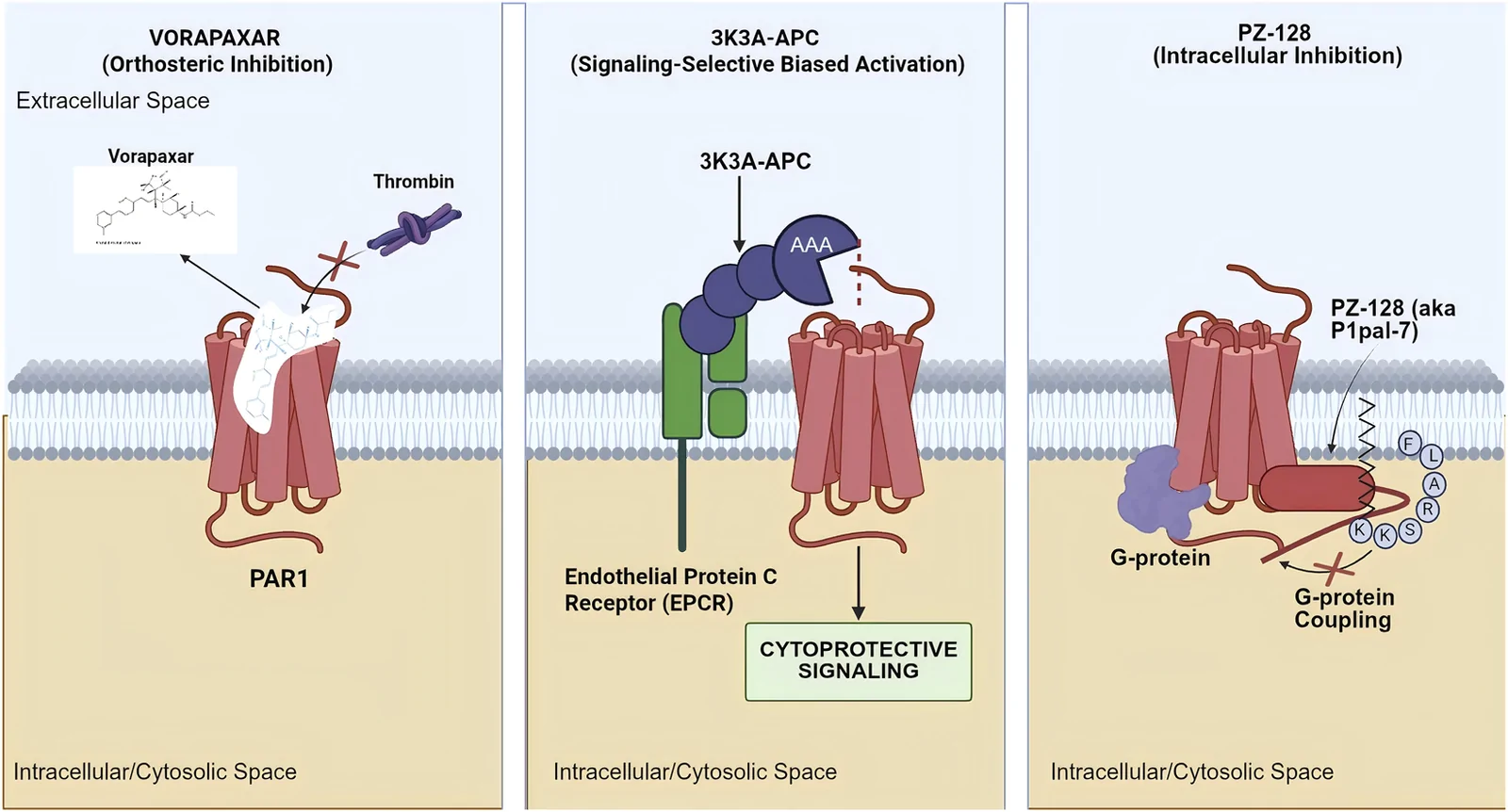
Mechanisms of action of representative PAR1 modulators. This figure summarizes three classes of PAR1 modulators: the orthosteric antagonist vorapaxar, the biased agonist 3K3A-APC, and the intracellular inhibitor PZ-128. Vorapaxar binds the transmembrane ligand-binding pocket and blocks thrombin-induced receptor activation. 3K3A-APC binds EPCR and cleaves PAR1 at a non-canonical site to trigger cytoprotective biased signaling with reduced anticoagulant activity. PZ-128 inhibits PAR1 from the intracellular side by blocking receptor-G protein coupling, thereby reversibly suppressing downstream signaling and platelet aggregation.

DM is a well-established risk factor for coronary heart disease, ischemic stroke, and other vascular diseases (Sarwar et al., 2010). As the principal receptor mediating thrombin-induced platelet activation, PAR1 is broadly involved in thrombosis, inflammation, and tissue remodeling, and its dysregulation has been linked to cardiovascular disease, neurological injury, and cancer (Table 2). Owing to its distinctive signaling properties, PAR1 has emerged as an attractive therapeutic target. Antagonists, biased agonists, and intracellular inhibitors targeting PAR1 have shown therapeutic potential in both preclinical and clinical studies, supporting its relevance to the management of DM and its complications (Figure 4).

**TABLE 2:** Representative PAR1-targeted drugs and their applications in diabetes mellitus and its complications.

Drug name	Drug type	Mechanism of action	Application/potential benefit	Key advantages	Limitations
Vorapaxar	Classical PAR1 antagonist (oral)	Highly selectively antagonizes PAR1, blocking thrombin-induced platelet activation without interfering with the ADP/collagen pathway	① Secondary cardiovascular prevention in DM patients: reduces the risk of major cardiovascular events, with more pronounced absolute benefit in high-ischemic-risk populations ② DKD protection: inhibits mesangial cell proliferation and ECM accumulation, reduces proteinuria, and lowers cystatin C levels	First approved PAR1 antagonist; complementary to aspirin and P2Y12 inhibitors	Increased bleeding risk Exclude populations with a history of stroke/TIA; limits long-term application in chronic diseases
3K3A-APC	Biased agonist (engineered APC mutant)	Cleaves PAR1 at the non-canonical Arg46 site, preserving cytoprotective signaling; >90% loss of anticoagulant activity	① Diabetic atherosclerosis: promotes inflammation resolution via MerTK/Arg1, restores macrophage function through Rac1-ATF6α activation, and attenuates plaque inflammation ② DKD protection: inhibits podocyte apoptosis via the EPCR-PAR1-Bax/mitochondrial pathway and maintains the filtration barrier ③ Other potential applications: ischemic stroke, multiple sclerosis, multi-organ ischemia/reperfusion injury, etc	Avoids bleeding risk; pleiotropic protection (anti-inflammatory, anti-apoptotic, tissue repair)	Immunogenicity; efficacy in complex disease models remains to be validated
PZ-128	Intracellular inhibitor (lipopeptide)	Inserts into the inner leaflet of the membrane, mimicking the “off” state of PAR1, reversibly blocking G-protein coupling, and inhibiting platelet aggregation	① Perioperative antithrombotic therapy in DM patients with cardiovascular disease (e.g., PCI): synergizes with aspirin/clopidogrel, inhibiting platelet aggregation in a dose-dependent manner ② Potential pleiotropic effects such as tumor suppression	Rapid onset, short half-life; does not affect coagulation function, low bleeding risk; reversible inhibition	Long-term safety and efficacy of combination therapy require further clarification The efficacy of combination with different targeted therapies remains to be validated

### 5.1 PAR1 orthosteric antagonist

Since PAR1 mediates thrombin-dependent platelet activation, the development of antithrombotic agents targeting this receptor has long been a focus of cardiovascular research. Among the PAR1-directed antithrombotic drugs, vorapaxar is the most advanced (Künze and Isermann, 2023). Vorapaxar, a highly specific oral PAR1 antagonist developed by Merck, was the first drug in its class to receive global approval. By antagonizing PAR1, vorapaxar blocks thrombin-induced platelet activation without interfering with ADP- or collagen-mediated platelet aggregation, thereby reducing clinical thrombotic events and complementing traditional antiplatelet agents such as aspirin and P2Y12 inhibitors. Clinical studies have shown that vorapaxar is particularly effective for secondary cardiovascular prevention in patients with DM, further reducing the risk of major cardiovascular events on top of standard therapy, with greater absolute benefit in patients at high ischemic risk. This mechanism offers an additional option for secondary prevention in patients with DM (Cavender et al., 2015). However, an increased bleeding risk remains its principal safety concern, which imposes certain limitations on clinical application (Poole and Elkinson, 2014). In the TRA 2°P-TIMI 50 trial, Cavender et al. showed that vorapaxar provided a favorable net clinical benefit in patients with diabetes and prior myocardial infarction but significantly increased bleeding risk. This risk was amplified by concomitant thienopyridine use, advanced age, obesity, and renal insufficiency. Because patients with a history of stroke or TIA were excluded, clinical use of vorapaxar requires individualized benefit-risk assessment and careful bleeding risk management (Cavender et al., 2015).

In addition to its cardiovascular benefits, vorapaxar has shown renoprotective potential in diabetic kidney disease. In a streptozotocin-induced diabetic mouse model, vorapaxar did not affect blood glucose levels but inhibited PAR1 signaling, reduced mesangial cell proliferation and extracellular matrix accumulation, and consequently decreased proteinuria, mesangial expansion, fibronectin deposition, and plasma cystatin C levels (Waasdorp et al., 2018). These findings support PAR1 as a potential therapeutic target in DKD. It should be noted, however, that vorapaxar may increase bleeding risk; therefore, the balance between renal protection and bleeding risk must be weighed in its application. In summary, although classical PAR1 antagonism has established antithrombotic efficacy, its bleeding risk remains a major limitation in the long-term management of chronic diseases such as DM, highlighting the need for safer PAR1-targeted strategies.

### 5.2 Drugs developed on the basis of PAR1 biased signaling

Although PAR1 orthosteric inhibitors have been developed, their use is accompanied by a concomitant increase in bleeding risk. Beyond classical antagonists, modulators developed based on PAR1 biased signaling have also shown promise, with 3K3A-APC and Parmodulins being a representative example. 3K3A-APC is a genetically engineered APC variant generated by site-directed mutagenesis in which three lysine residues are replaced with alanine, resulting in a signaling-selective mutant. This variant retains cellular signaling activity while losing more than 90% of its anticoagulant activity. By cleaving PAR1 at the non-canonical Arg46 site, it induces biased signaling and exerts cytoprotective effects (Griffin et al., 2018).

In diabetic macrovascular disease, 3K3A-APC mimics the cytoprotective actions of APC. It upregulates Mer receptor tyrosine kinase (MerTK) and maintains arginase-1 (Arg1) homeostasis, thereby promoting resolution of inflammation through Arg1-mediated arginine metabolism. It also activates the Rac1–activating transcription factor-6 alpha (ATF6α) signaling axis, restores Rac1 activity impaired by high glucose, and suppresses excessive ATF6α cleavage, thereby alleviating endoplasmic reticulum stress, improving macrophage cytoskeletal remodeling and efferocytosis, and reducing secondary necrosis and inflammatory injury within plaques (Ambreen et al., 2025). These findings suggest that 3K3A-APC may represent a promising therapeutic strategy for diabetes-associated atherosclerosis. In DKD, APC inhibits the p53/Bax pathway and mitochondrial apoptosis via the EPCR–PAR1–Bax/mitochondrial axis, thereby protecting glomerular podocytes and endothelial cells to preserve the integrity of the filtration barrier while reducing oxidative stress (Isermann et al., 2007). These findings further suggest that 3K3A-APC may exert renoprotective effects in DKD and support PAR1 biased signaling as a potential therapeutic target in diabetic kidney disease. Owing to its anti-inflammatory, anti-apoptotic, and tissue-repair properties, 3K3A-APC has shown therapeutic potential in several disease models, including ischemic stroke, multiple sclerosis, and multi-organ ischemia/reperfusion injury. Its potential application may extend to a broader range of diseases, including DM and its complications, either as monotherapy or in combination therapy. However, its clinical translation still faces challenges, including potential immunogenicity and the need to validate efficacy in complex disease settings (Griffin et al., 2018; Wang et al., 2016).

However, several obstacles limit the clinical translation of these drugs developed on the basis of PAR1 biased signaling. Early-generation compounds showed notable off-target effects, and optimized derivatives have not yet achieved sufficient pathway selectivity. The precise PAR1 binding sites and allosteric mechanisms remain poorly defined, partly because high-resolution structural evidence is lacking. Species differences in platelet receptors between humans and experimental animals further limit the extrapolation of preclinical findings. In addition, long-term toxicological data remain scarce, leaving potential risks related to endothelial homeostasis and embryonic development insufficiently assessed. Several lead compounds also show poor plasma stability and unfavorable pharmacokinetic profiles. Together, these limitations have delayed the clinical development of this drug class (Künze and Isermann, 2023).

In summary, these drugs may balance antithrombotic activity and cytoprotection through allosteric modulation of PAR1 signaling. Their main advantage is the selective inhibition of pathological signaling, such as thrombosis-related Gαq activation, while preserving protective pathways, including aPC-mediated endothelial signaling. However, their clinical utility requires further validation. Future PAR1-targeted drug development may thus increasingly focus on biased allosteric modulators to address current translational barriers.

### 5.3 Intracellular PAR1 inhibitor

PZ-128, a novel intracellular PAR1 inhibitor, represents a new approach to PAR1-targeted drug development. PZ-128 is a cell-penetrating lipopeptide derived from the juxtamembrane region of the third intracellular loop of PAR1, with enhanced membrane-crossing capacity conferred by N-terminal palmitoylation. Its structure is designed to mimic the “off” state of PAR1. By inserting into the inner leaflet of the cell membrane, PZ-128 specifically blocks receptor–G protein coupling and reversibly inhibits PAR1-mediated platelet aggregation. In clinical studies, PZ-128 acts rapidly, inhibits platelet aggregation in a dose-dependent manner, and shows synergy with aspirin and clopidogrel without affecting coagulation or cardiac electrical activity, suggesting a more favorable safety profile than traditional agents (Zhang et al., 2012; Gurbel et al., 2016). Compared with extracellular inhibitors, PZ-128 has a short half-life and fewer off-target effects, which may reduce bleeding risk and avoid the limitations of irreversible inhibition. It may therefore be particularly useful in peri-PCI management and as an adjunct to existing antiplatelet therapies. This feature is of particular clinical value in patients with DM and concomitant cardiovascular disease. PAR1 also plays an oncogenic role in cancer progression by promoting invasion, metastasis, and angiogenesis through both canonical and non-canonical pathways. Accordingly, PZ-128 may inhibit tumor growth, metastasis, and angiogenesis by targeting PAR1-related signaling, suggesting broader therapeutic potential. However, the long-term safety of PZ-128 and its efficacy in combination with other targeted therapies remain to be clarified, despite its advantages in target specificity and reversibility (Covic and Kuliopulos, 2018). In DM, PZ-128 is currently best positioned as a reversible antithrombotic option with potentially lower bleeding risk, although further studies are needed to define its long-term safety and combination strategies for cardiovascular risk management.

In conclusion, PAR1 has emerged as a versatile target in drug development. Existing agents have shown therapeutic potential in diseases commonly associated with DM, including cardiovascular disease, neurological injury, and cancer. However, there is currently no definitive evidence that existing glucose-lowering agents improve DM or its complications by modulating PAR1 signaling, indicating an important area for future research. The clinical translation of PAR1-targeted therapies for DM and its complications also faces several barriers, including bleeding risk, species differences in receptor expression between experimental animals and humans, limited predictability of drug efficacy, and challenges related to drug delivery and pharmacokinetics. Future efforts should focus on structural optimization, development of PAR1-biased allosteric ligands, and rational combination strategies. These approaches may help overcome current translational barriers and support the development of safer and more effective PAR1-targeted therapies for DM and its chronic complications.

## 6 Conclusion and perspectives

In summary, PAR1 serves as a key molecular hub linking metabolic dysregulation, hypercoagulability, and chronic inflammation in diabetes. Through its biased signaling properties, PAR1 exerts context-dependent and bidirectional regulatory effects and participates in the development and progression of multi-organ diabetic complications. PAR1 signals through multiple G protein- and β-arrestin-mediated pathways. On the one hand, aberrant PAR1 activation facilitates inflammatory activation, fibrosis, vascular injury, and neural damage, thereby contributing to diabetic kidney disease, diabetic retinopathy, diabetic peripheral neuropathy, and diabetic cardiovascular disease. On the other hand, PAR1 can also function as an endogenous protective receptor by exerting anti-inflammatory and anti-apoptotic effects and maintaining endothelial barrier integrity. This dual functionality makes PAR1 a promising therapeutic target for diabetic complications.

Several PAR1-targeted strategies have been developed, including vorapaxar as an orthosteric antagonist, 3K3A-APC as a biased agonist, parmodulins/ML161 as allosteric modulators, and PZ-128 as an intracellular inhibitor. These agents have shown cardioprotective, renoprotective, and neuroprotective effects in preclinical studies, suggesting translational potential. However, several limitations remain. Current experimental models do not fully recapitulate the complex *in vivo* microenvironment of T2DM. The interactions among PAR family members and the cell type-specific functions of PAR1 remain incompletely defined. Most importantly, species differences, bleeding risks associated with systemic PAR1 targeting, a lack of tissue-specific regulatory approaches, and insufficient evidence regarding the long-term safety and clinical efficacy of current PAR1-targeted agents continue to hinder clinical translation.
